# Supplementary material for: A short linear motif in scaffold Nup145C connects Y-complex with pre-assembled outer ring Nup82 complex
Source: Nat Commun. 2017 Oct 24;8:1107. doi: 10.1038/s41467-017-01160-9 (PMC5653651; doi:10.1038/s41467-017-01160-9)
Supplement: Supplementary file 1 — Supplementary Information [file 41467_2017_1160_MOESM1_ESM.pdf]

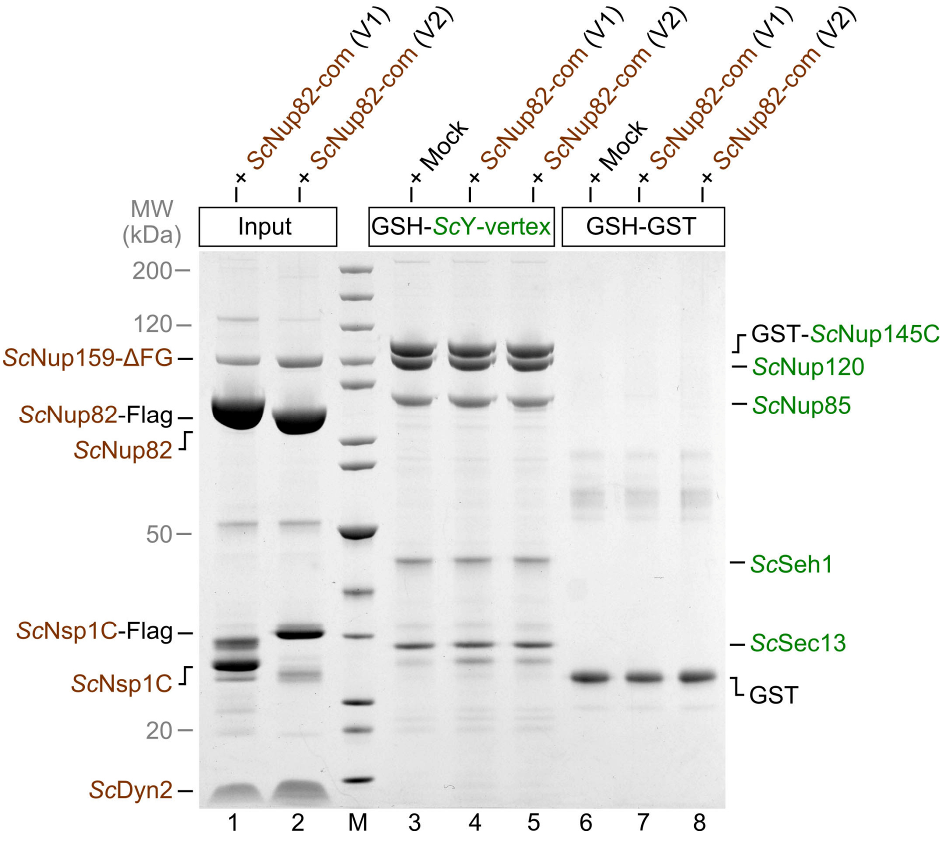

Supplementary Figure 1 | *In vitro* binding assay reveals no stable interaction between yeast *Sc*Nup82 complex and *Sc*Y-vertex. Immobilized yeast *Sc*Y-vertex (GST-*Sc*Nup145C–Sec13–Nup120–Nup85–Seh1) was incubated with soluble yeast *Sc*Nup82 complex (*Sc*Dyn2–Nup159-ΔFG–Nsp1C–Nup82), which was affinity-purified via *Sc*Nup82-Flag-TEV-ProtA (V1) or *Sc*Nsp1C-Flag-TEV-ProtA (V2). After the binding reaction, the final SDS-eluates were analyzed by SDS-PAGE and Coomassie staining. No significant binding of *Sc*Nup82 complex to *Sc*Y-vertex was observed. The experiment was performed twice with consistent results. M, molecular weight marker bands (indicated in grey at the left); Mock, purification buffer with *E. coli* whole cell lysate. An uncropped image of the gel is shown in Supplementary Fig. 4g.

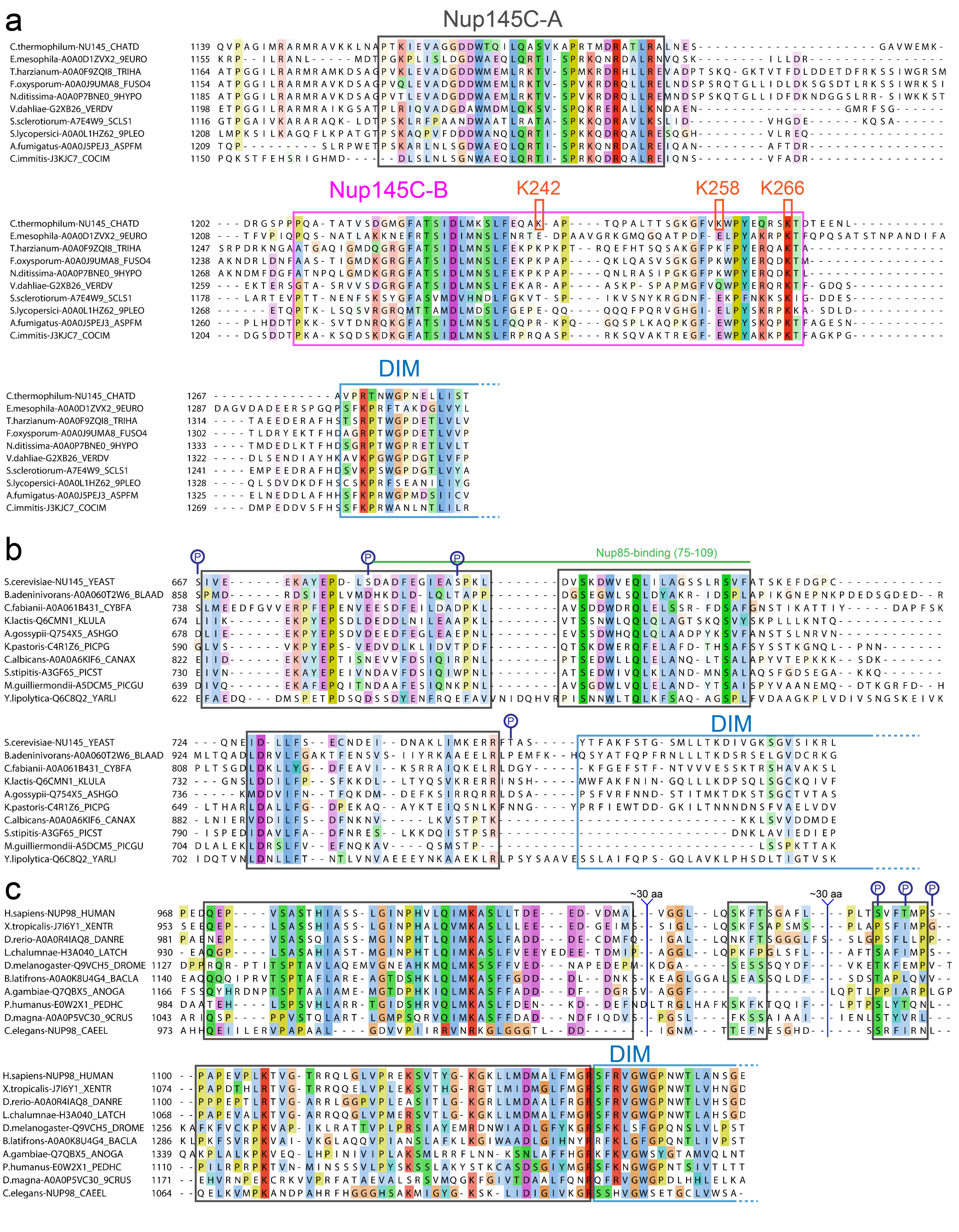

Supplementary Figure 2 | Multiple sequence alignments of Nup145C from different organisms. Alignments were restricted to orthologs of different clades, i.e. Pezizomycotina (**a**, including *C. thermophilum*), Saccharomycotina (**b**, including *S. cerevisiae*), and Metazoa (**c**, including *H. sapiens*). Conserved blocks are marked by boxes. The crosslinked lysine residues of *Ct*Nup145C (K242, K258, and K266) are marked by orange, vertical boxes. DIM, domain invasion motif. Encircled letters ‘P’ mark phosphorylation sites[1-5].

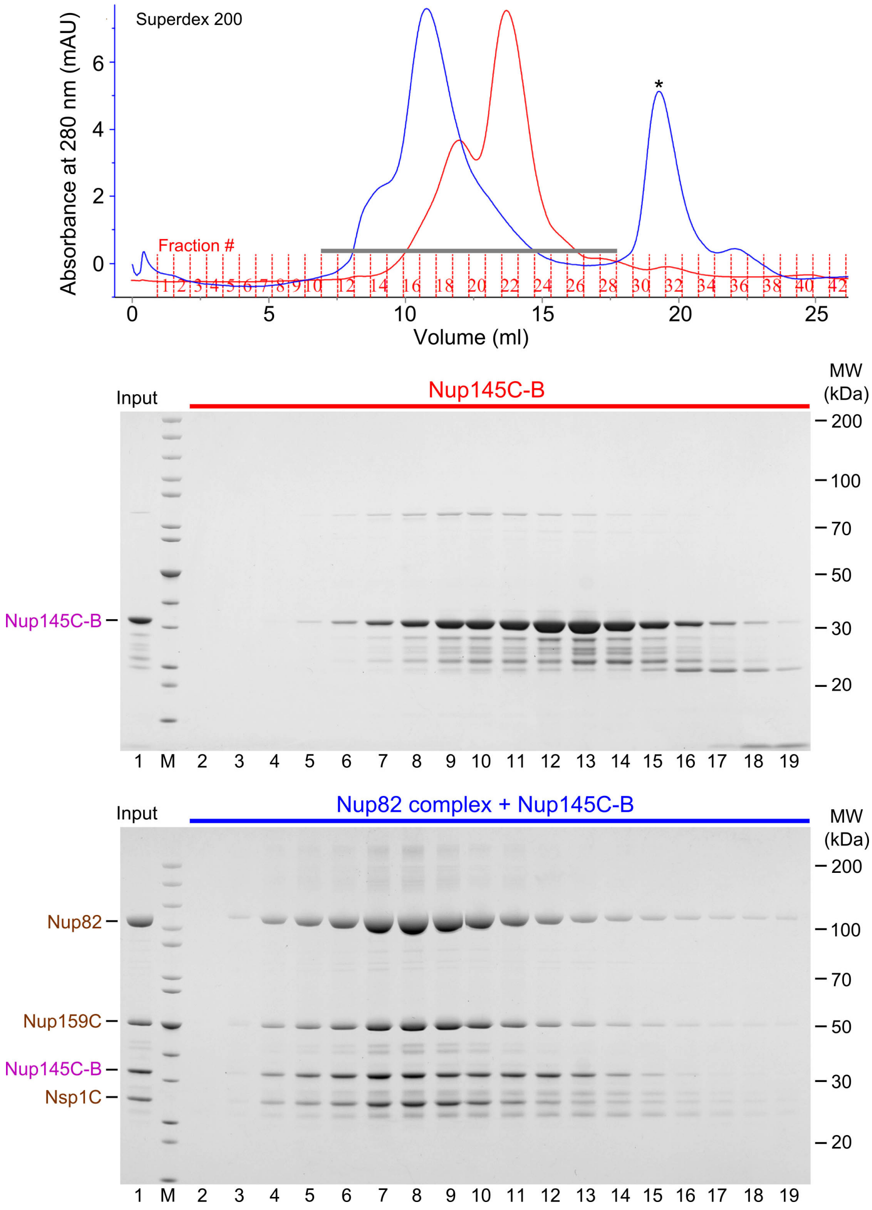

Supplementary Figure 3 | Gel filtration chromatography reveals robust binding of *Ct*Nup145C-B motif (82CIM) to the *Ct*Nup82 complex. Top, Elution profile (recorded at 280 nm) of the purified *Ct*Nup145C-B (red) and *in vitro* reconstituted *Ct*Nup82–Nup159C–Nsp1C–Nup145C-B complex (blue) during size exclusion chromatography (SEC). The asterisk marks the elution peak of the Flag peptide used for the elution of the complex. The grey bar indicates SEC fractions that were subsequently analyzed by SDS-PAGE and Coomassie staining (middle and lower panels). Note that the *Ct*Nup145C-B motif remains associated with the *Ct*Nup82 complex during SEC. The experiment was performed twice with consistent results. AU, arbitrary units; M, marker; MW, molecular weight. Uncropped images of the gels are shown in Supplementary Fig. 4h,i.

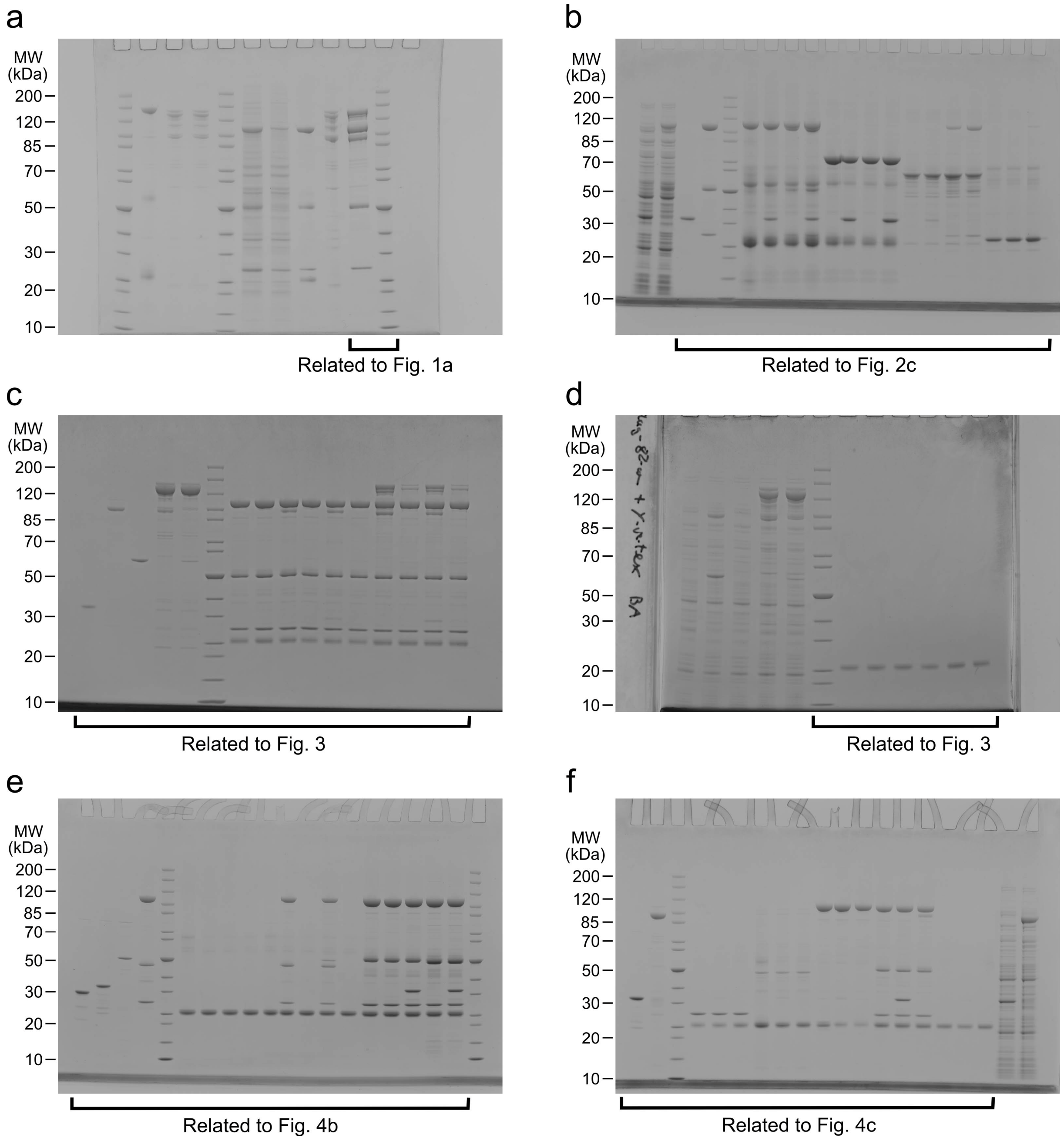
a
MW (kDa)
200
120
85
70
50
30
20
10
Related to Fig. 1a
b
MW (kDa)
200
120
85
70
50
30
20
10
Related to Fig. 2c
c
MW (kDa)
200
120
85
70
50
30
20
10
Related to Fig. 3
d
MW (kDa)
200
120
85
70
50
30
20
10
Related to Fig. 3
e
MW (kDa)
200
120
85
70
50
30
20
10
Related to Fig. 4b
f
MW (kDa)
200
120
85
70
50
30
20
10
Related to Fig. 4c

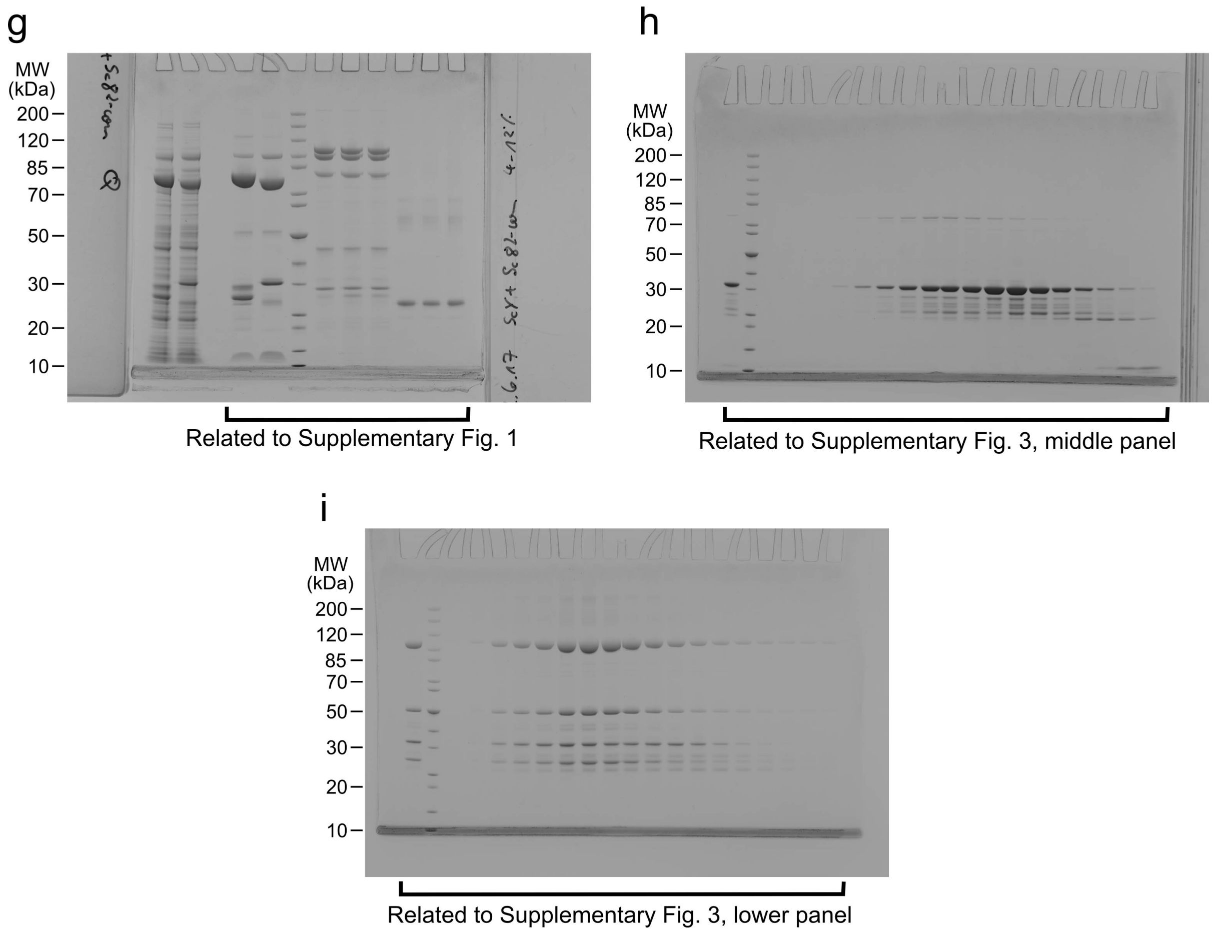

Supplementary Figure 4 | Uncropped gel images. Uncropped images of Coomassie-stained SDS-PAGE gels shown in the indicated Figures. MW, molecular weight.

Supplementary Table 1 | Plasmids used in this study.

Plasmid	Relevant information	References
pPROEX-GST-TEV-*Ct*Nup145N-ΔFG (aa606-993)	Trc promoter, pBR322 origin, AmpR	6
pET24b-His-TEV-*Ct*Sec13	f1 origin, T7 promoter, pBR322 origin, KanR2	This study
pET24b-GST-TEV-*Ct*Nup145C-NTD (aa1-270)	f1 origin, T7 promoter, pBR322 origin, KanR2	This study
pET24b-GST-TEV-*Ct*Nup145C-A-Flag (aa163-195)	f1 origin, T7 promoter, pBR322 origin, KanR2	This study
pET24b-GST-TEV-*Ct*Nup145C-A-His (aa163-195)	f1 origin, T7 promoter, pBR322 origin, KanR2	This study
pET24b-GST-TEV-*Ct*Nup145C-B-Flag (aa215-270)	f1 origin, T7 promoter, pBR322 origin, KanR2	This study
pET24b-GST-TEV-*Ct*Nup145C-B-His (aa215-270)	f1 origin, T7 promoter, pBR322 origin, KanR2	This study
pET24b-GST-TEV-*Ct*Nsp1C-Flag (aa448-678)	f1 origin, T7 promoter, pBR322 origin, KanR2	This study
pET24b-GST-TEV-*Ct*Nup159C-Flag (aa1102-1481)	f1 origin, T7 promoter, pBR322 origin, KanR2	This study
pPROEXHT-GST-TEV-*Sc*Nup145C-*Sc*Sec13-T7-*Sc*Nup120	Trc promoter, T7 promoter, pBR322 origin, AmpR	This study
pET24d-*Sc*Nup85-*Sc*Seh1	f1 origin, T7 promoter, pBR322 origin, KanR2	7
YEplac112-ProtA-TEV-*Ct*Nup82-Flag	2μ, p*GAL1-10*, TRP1, AmpR	This study
YEplac112-*Ct*Nsp1C-His (aa448-678)	2μ, p*GAL1-10*, TRP1, AmpR	6
YEplac181-Flag-*Ct*Nup159C (aa1102-1481)	2μ, p*GAL1-10*, LEU2, AmpR	6
YEplac181-Flag-TEV-*Ct*Nup159C (aa1102-1481)	2μ, p*GAL1-10*, LEU2, AmpR	6
YEplac195-*Ct*Nup82	2μ, p*GAL1-10*, URA3, AmpR	6
YEplac181-P2-*Ct*Nup120-P1-ProtA-TEV-His-*Ct*Nup85	2μ, p*GAL1-10*, LEU2, AmpR	8
YEplac112-*Ct*Nup145C	2μ, p*GAL1-10*, TRP1, AmpR	8
YEplac112-ProtA-TEV-*Ct*Nup145C	2μ, p*GAL1-10*, TRP1, AmpR	8
YEplac112-*Ct*Nup145C-CTD (aa271-800)	2μ, p*GAL1-10*, TRP1, AmpR	This study
YEplac112-*Ct*Nup145C-CTD-TEV-ProtA (aa271-800)	2μ, p*GAL1-10*, TRP1, AmpR	This study
YEplac181-P2-*Sc*Nsp1C(aa573-823)-P1-*Sc*Nup82-Flag-TEV-ProtA	2μ, p*GAL1-10*, LEU2, AmpR	9
YEplac195-P2-*Sc*Dyn2-P1-*Sc*NUP159-ΔFG (Δaa459-1083)	2μ, p*GAL1-10*, URA3, AmpR	9
YEplac181-P2-*Sc*Nsp1C(aa573-823)-Flag-TEV-ProtA-P1-*Sc*Nup82	2μ, p*GAL1-10*, LEU2, AmpR	9

Supplementary Table 2 | List of medium (ld-score at least 25) and high (ld-score at least 30) confidence crosslinks identified in this work. Please see Supplementary Data 1 for a complete list of crosslinks, mono-links, and additional attributes and scores of the detected crosslinks.

Crosslinked peptides	Protein1	Protein2	AbsPos1	AbsPos2	ld-Score	fdr
IKSFAPAWLNEPAPGHK-AKEEDLR-a2-b2	Nup82	Nup159	5	1249	44.95	0
SKTDTEENLAVPR-KQYQQELDR-a2-b1	Nup145C	Nup120	266	1228	42.68	0
VGDDTEESTPASQMPSLKIPSEIRK-KQYQQELDR-a18-b1	Nup82	Nup120	845	1228	41.75	0
SKTDTEENLAVPR-LEEIKTLR-a2-b5	Nup145C	Nup82	266	812	41.03	0
KDSSDFDDGAATAGYR-AKEEDLRK-a1-b2	Nup82	Nup159	92	1249	40.32	0
IIKTPVADDIR-TYKLAEK-a3-b3	Nup82	Nsp1	110	576	40.58	0
LEEIKTLR-DLAKMIK-a5-b4	Nup82	Nsp1	812	594	41.8	0
KDSSDFDDGAATAGYR-IQKLYTSTYEAER-a1-b3	Nup82	Nsp1	92	508	39.4	0
LAEKLTDQLDEMGK-SKTDTEENLAVPR-a4-b2	Nsp1	Nup145C	580	266	38.96	0
GTNDEEYSQTGSKR-SKTDTEENLAVPR-a13-b2	Nup120	Nup145C	1149	266	37.53	0
GLLAATGTPSPTKK-DIYVIGEKPR-a13-b8	Nup159	Nup82	1435	398	37.38	0
LAEKLTDQLDEMGK-IIKTPVADDIR-a4-b3	Nsp1	Nup82	580	110	35.87	0
CKGAAAASDPR-MQKFSTAVR-a2-b3	Nup145C	Nup120	769	1203	36.51	0
SLFEQAKAPTQPALTTSGK-TINKMTSMAEKR-a7-b4	Nup145C	Nup159	242	1339	35.62	0
AKEEDLRK-TYKLAEK-a2-b3	Nup159	Nsp1	1249	576	35.62	0
IKSFAPAWLNEPAPGHK-DLAKLR-a2-b4	Nup82	Nup159	5	1245	35.25	0
SSVGPTSTKK-DLAKLR-a9-b4	Nup82	Nup159	91	1245	34.62	0
KARPSSSSEEDQQLLER-SKTDTEENLAVPR-a1-b2	Nup120	Nup145C	1184	266	34.26	0
IHTITGDDLSDDPPVSEDQLIKQR-IKDVEGTMAAIHNLGR-a22-b2	Nup82	Nup159	734	1227	33.57	0
KARPSSSSEEDQQLLER-KHLPNWER-a1-b1	Nup120	Nup145C	1184	702	33.52	0
TINKMTSMAEKR-GFVKWPYEQR-a4-b4	Nup159	Nup145C	1339	258	33.3	0
SSLSSSSSSRPASATADDEKGYR-KVNEVK-a20-b1	Nup120	Nup82	58	705	33.15	0

Crosslinked peptides	Protein1	Protein2	AbsPos1	AbsPos2	Id-Score	fdr
AKEEDLRK-KIKPGPR-a2-b1	Nup159	Nup82	1249	40	32.64	0
TTTRELSDKEK-AKEEDLRK-a9-b2	Nup82	Nup159	769	1249	35.75	0
SSVGPTSTKK-TYKLAEK-a9-b3	Nup82	Nsp1	91	576	32.22	0
TYKLAEK-DLAKLR-a3-b4	Nsp1	Nup159	576	1245	31.71	0
SSVGPTSTKKDSSDFDDGAATAGYR-AKEEDLRK-a9-b2	Nup82	Nup159	91	1249	37.3	0
SSVGPTSTKKDSSDFDDGAATAGYR-AKLASANAAR-a10-b2	Nup82	Nup159	92	1309	30.85	0
SKTDTEENLAVPR-DLAKMIK-a2-b4	Nup145C	Nsp1	266	594	30.56	0
LLDSESAGDKK-KHLPNWER-a10-b1	Nup120	Nup145C	1183	702	30.38	0
SLFEQAKAPTQPALTTSGK-TINKMTSMAEKR-a7-b11	Nup145C	Nup159	242	1346	32.02	0.034
LLTEAEEALTVLKAK-SKTDTEENLAVPR-a13-b2	Nup159	Nup145C	1307	266	29.57	0
IKDVEGTMAAIHNLGR-IQKLYTSTYEAER-a2-b3	Nup159	Nsp1	1227	508	36.54	0
ELDELYAKQMGSAAGEQAAGPDQERER-SSVGPTSTKK-a8-b9	Nsp1	Nup82	554	91	28.92	0
IIKTPVADDIR-AKEEDLR-a3-b2	Nup82	Nup159	110	1249	33.95	0
SSVGPTSTKKDSSDFDDGAATAGYR-TYKLAEK-a10-b3	Nup82	Nsp1	92	576	28.23	0
LFNAQVDPDQIALTKALPLSAEQLAQQNELR-KHHSSPR-a15-b1	Nup159	Nup120	1270	75	28.23	0
IQKLYTSTYEAER-IIKTPVADDIR-a3-b3	Nsp1	Nup82	508	110	28.23	0
TINKMTSMAEKR-GFVKWPYEQR-a11-b4	Nup159	Nup145C	1346	258	27.94	0
SKTDTEENLAVPR-TINKMTSMAEK-a2-b4	Nup145C	Nup159	266	1339	27.81	0
IQKLYTSTYEAER-SSVGPTSTKK-a3-b9	Nsp1	Nup82	508	91	32.35	0
SSVGPTSTKK-EFKEQAAK-a9-b3	Nup82	Nsp1	91	486	31.25	0.032
SLFEQAKAPTQPALTTSGK-LLTEAEEALTVLKAK-a7-b13	Nup145C	Nup159	242	1307	27.29	0
VAAAQKAAGSMGANVPGTETDAAESFYR-KHHSSPR-a6-b1	Nsp1	Nup120	649	75	25.91	0.017
SKTDTEENLAVPR-LLDSESAGDKK-a2-b10	Nup145C	Nup120	266	1183	25.51	0.017
AKEEDLRK-KVNEVKNR-a2-b6	Nup159	Nup82	1249	710	25.3	0.017
YQKEFKEQAAK-SSVGPTSTKK-a3-b9	Nsp1	Nup82	483	91	27.81	0.023
IIKTPVADDIR-DLAKLR-a3-b4	Nup82	Nup159	110	1245	39.76	0
SKTDTEENLAVPR-IIKTPVADDIR-a2-b3	Nup145C	Nup82	266	110	39.1	0

Crosslinked peptides	Protein1	Protein2	AbsPos1	AbsPos2	Id-Score	fdr
AKEEDLRK-KIKPGPR-a2-b3	Nup159	Nup82	1249	42	30.76	0.031
KLFNAQVDPDQIALTK-KHHSSPR-a1-b1	Nup159	Nup120	1255	75	28.71	0.026
VAAAQKAAGSMGANVPGTETDAAESFYR-TTTRELSDKEK-a6-b9	Nsp1	Nup82	649	769	28.46	0.025
IHTITGDDLSDDPPVSEDQLIKQR-AKEEDLR-a22-b2	Nup82	Nup159	734	1249	26.71	0.02
LSMYTAEEKRELR-KVNEVK-a9-b1	Nup159	Nup82	1446	705	25.78	0.019
VPDDSILSSSPAPSTPDKSRR-SKTDTEENLAVPR-a18-b2	Nup85	Nup145C	21	266	25.43	0.019
TINKMTSMAEKR-MRAVKK-a4-b5	Nup159	Nup145C	1339	160	25	0.019
AKEEDLR-DLAKLR-a2-b4	Nup159	Nup159	1249	1245	43.38	0
IKDVEGTMAAIHNLGR-DLAKLR-a2-b4	Nup159	Nup159	1227	1245	41.32	0
IIKTPVADDIR-KVNEVK-a3-b1	Nup82	Nup82	110	705	40.68	0
KQYQQELDR-MQKFSTAVR-a1-b3	Nup120	Nup120	1228	1203	40.4	0
KARPSSSSEEDQQLLER-MQKFSTAVR-a1-b3	Nup120	Nup120	1184	1203	40.22	0
RGTEIFVACGKQIR-KIKPGPR-a11-b1	Nup82	Nup82	62	40	39.46	0
SKTDTEENLAVPR-GFVKWPYEQR-a2-b4	Nup145C	Nup145C	266	258	38.51	0
TKNLATASSIYYR-GLGTYKETVSR-a2-b6	Nup120	Nup120	63	486	39.59	0
LLDSESAGDKK-MQKFSTAVR-a10-b3	Nup120	Nup120	1183	1203	36.94	0
GLLAATGTPSPTKK-KLSMYTAEEK-a13-b1	Nup159	Nup159	1435	1437	36.72	0
EQAAKVMEWDR-YQKEFK-a5-b3	Nsp1	Nsp1	491	483	37.66	0
LTDQLDEMGKDLAK-MIKEINDMSNTLSK-a10-b3	Nsp1	Nsp1	590	597	39.2	0
GLLAATGTPSPTKKK-LSMYTAEEKRELR-a14-b9	Nup159	Nup159	1436	1446	35.97	0
TKNLATASSIYYR-SGSSSPTLSKR-a2-b10	Nup120	Nup120	63	786	34.74	0
KKDEPVR-GKVQEK-a1-b2	Nup85	Nup85	171	166	35.5	0
GTNDEEYSQTGSKR-KQYQQELDR-a13-b1	Nup120	Nup120	1149	1228	37.5	0
SGKGIAESICSVIGSTTTLDR-GLGTYKETVSR-a3-b6	Nup120	Nup120	494	486	33.99	0
KRSATEGGLGDASR-TELSVSPSKSR-a1-b9	Nup120	Nup120	161	774	33.9	0
RGTEIFVACGKQIR-IKPGPR-a11-b2	Nup82	Nup82	62	42	36.95	0
RGTEIFVACGKQIR-IIKTPVADDIR-a11-b3	Nup82	Nup82	62	110	36.9	0

Crosslinked peptides	Protein1	Protein2	AbsPos1	AbsPos2	Id-Score	fdr
KIKPGPR-KVNEVK-a1-b1	Nup82	Nup82	40	705	33.4	0
KDSSDFDDGAATAGYR-IIKTPVADDIR-a1-b3	Nup82	Nup82	92	110	41.57	0
SSLSSSSSSRPASATADDEKGYR-TKNLATASSIYYR-a20-b2	Nup120	Nup120	58	63	35.84	0
IIKTPVADDIR-SSVGPTSTKK-a3-b9	Nup82	Nup82	110	91	34.65	0
KLFNAQVDPDQIALTK-LRAKEEDLR-a1-b4	Nup159	Nup159	1255	1249	32.81	0
GLLAATGTPSPTKK-LSMYTAEEKR-a13-b9	Nup159	Nup159	1435	1446	33.04	0
KIKPGPRR-KVNEVK-a3-b1	Nup82	Nup82	42	705	33	0
IIKTPVADDIR-ELSDKEK-a3-b5	Nup82	Nup82	110	769	32.09	0
KLFNAQVDPDQIALTK-DLAKLR-a1-b4	Nup159	Nup159	1255	1245	31.85	0
SSLSSSSSSRPASATADDEKGYR-KHHSSPR-a20-b1	Nup120	Nup120	58	75	31.77	0
LFAPAADDGTATVPLAYGKK-IKPGPRR-a19-b2	Nup82	Nup82	39	42	32.06	0
KRSATEGGLGDASR-QEKVADAPLILNLR-a1-b3	Nup120	Nup120	161	106	31.45	0
VADVCRQEKVADAPLILNLR-TELSVSPSKSR-a9-b9	Nup120	Nup120	106	774	35.05	0
SSVGPTSTKK-KIKPGPR-a9-b3	Nup82	Nup82	91	42	34.06	0
CEALQGELRDQIKK-VNEVKNR-a13-b5	Nup82	Nup82	704	710	30.48	0
SSVGPTSTKK-KVNEVK-a9-b1	Nup82	Nup82	91	705	30.87	0.043
SLFEQAKAPTQPALTTSGK-SKTDTEENLAVPR-a7-b2	Nup145C	Nup145C	242	266	30.03	0
SLFEQAKAPTQPALTTSGK-GFVKWPYEQR-a7-b4	Nup145C	Nup145C	242	258	29.65	0
TKNLATASSIYYR-KHHSSPR-a2-b1	Nup120	Nup120	63	75	29.58	0
LLDSESAGDKK-KQYQQELDR-a10-b1	Nup120	Nup120	1183	1228	29.52	0
LFAPAADDGTATVPLAYGKK-GTEIFVACGKQIR-a19-b10	Nup82	Nup82	39	62	28.59	0
DPQEVGKWTVDPSQNNLIR-TGQILYTGDILNNTKR-a7-b15	Nup120	Nup120	314	306	33.77	0
IKSFAPAWLNEPAPGHK-SSVGPTSTKK-a2-b9	Nup82	Nup82	5	91	28.1	0
SIGVDDLEDEEEEEEKKK-KPQLLKPK-a16-b6	Nup85	Nup85	154	162	28.07	0
KDSSDFDDGAATAGYR-KIKPGPR-a1-b3	Nup82	Nup82	92	42	27.86	0
KRSATEGGLGDASR-SGSSSPTLSKR-a1-b10	Nup120	Nup120	161	786	27.74	0
KCEALQGELRDQIK-KVNEVK-a1-b1	Nup82	Nup82	691	705	27.6	0

Crosslinked peptides	Protein1	Protein2	AbsPos1	AbsPos2	Id-Score	fdr
GTNDEEYSQTGSKRK-MQKFSTAVR-a13-b3	Nup120	Nup120	1149	1203	26.56	0
SSVGPTSTKKDSSDFDDGAATAGYR-KVNEVK-a10-b1	Nup82	Nup82	92	705	26.22	0
LSMYTAEEKR-RKATLR-a9-b2	Nup159	Nup159	1446	1457	25.42	0
AKEEDLRK-AREAKR-a2-b5	Nup159	Nup159	1249	1455	25.37	0
LGKLNATSGEEDLDSRIEELAR-KQYQQELDR-a3-b1	Nup120	Nup120	1154	1228	31.37	0.024
KDEPVRGLFTGTSLAPPPLSK-GKVQEKK-a1-b6	Nup85	Nup85	172	170	25.14	0.047
AKEEDLRK-AKEEDLRK-a2-b2	Nup159	Nup159	1249	1249	35.16	0
KKDEPVR-GKVQEK-a2-b2	Nup85	Nup85	172	166	30.36	0.042
KSIGVDDLEDEEEEEEK-KNLFAAVSGER-a1-b1	Nup85	Nup85	138	105	38.63	0
KARPSSSSEEDQQLLER-KQYQQELDR-a1-b1	Nup120	Nup120	1184	1228	36.9	0
IDLLAKGQGSERDR-KVNEVK-a6-b1	Nup82	Nup82	543	705	36.31	0
NKTMDEIITR-WATDLAKYQK-a2-b7	Nsp1	Nsp1	465	480	35.55	0
FSKSTLETALAMYEK-GLGTYKETVSR-a3-b6	Nup120	Nup120	468	486	34.48	0
KSIGVDDLEDEEEEEEK-KKDEPVR-a1-b2	Nup85	Nup85	138	172	32.56	0
KSIGVDDLEDEEEEEEK-KKDEPVR-a1-b1	Nup85	Nup85	138	171	31.53	0.024
KKPQLLKPK-GKVQEK-a2-b2	Nup85	Nup85	157	166	31.23	0.047
GSKPADLLKK-MKGEDVIR-a3-b2	Nup85	Nup85	1082	1140	29.99	0.041
SIGVDDLEDEEEEEEKKK-KPQLLKPK-a17-b6	Nup85	Nup85	155	162	29.41	0.04
SSLSSSSSSRPASATADDEKGYR-STLETALAMYEKGLGTYK-a20-b12	Nup120	Nup120	58	480	29.4	0.04
IKSFAPAWLNEPAPGHK-KDSSDFDDGAATAGYR-a2-b1	Nup82	Nup82	5	92	28.93	0.038
VKANDQGSIHVTDCFPDAR-QEKVADAPLILNLR-a2-b3	Nup120	Nup120	354	106	28.36	0.037
KLFNAQVDPDQIALTK-GLLAATGTPSPTKK-a1-b13	Nup159	Nup159	1255	1435	25.46	0.049
SFAPAWLNEPAPGHKLFAPAADDGTATVPLAYGK-IIKTPVADDIR-a15-b3	Nup82	Nup82	20	110	25.35	0.048

Supplementary References

1. Li, X. et al. Large-scale phosphorylation analysis of alpha-factor-arrested Saccharomyces cerevisiae. *J Proteome Res* **6**, 1190-7 (2007).
2. Glavy, J.S. et al. Cell-cycle-dependent phosphorylation of the nuclear pore Nup107-160 subcomplex. *Proc Natl Acad Sci U S A* **104**, 3811-6 (2007).
3. Albuquerque, C.P. et al. A multidimensional chromatography technology for in-depth phosphoproteome analysis. *Mol Cell Proteomics* **7**, 1389-96 (2008).
4. Holt, L.J. et al. Global analysis of Cdk1 substrate phosphorylation sites provides insights into evolution. *Science* **325**, 1682-6 (2009).
5. Chi, A. et al. Analysis of phosphorylation sites on proteins from Saccharomyces cerevisiae by electron transfer dissociation (ETD) mass spectrometry. *Proc Natl Acad Sci U S A* **104**, 2193-8 (2007).
6. Fischer, J., Teimer, R., Amlacher, S., Kunze, R. & Hurt, E. Linker Nups connect the nuclear pore complex inner ring with the outer ring and transport channel. *Nat Struct Mol Biol* **22**, 774-81 (2015).
7. Lutzmann, M., Kunze, R., Buerer, A., Aebi, U. & Hurt, E. Modular self-assembly of a Y-shaped multiprotein complex from seven nucleoporins. *EMBO J* **21**, 387-97 (2002).
8. Thierbach, K. et al. Protein interfaces of the conserved Nup84 complex from Chaetomium thermophilum shown by crosslinking mass spectrometry and electron microscopy. *Structure* **21**, 1672-82 (2013).
9. Gaik, M. et al. Structural basis for assembly and function of the Nup82 complex in the nuclear pore scaffold. *J Cell Biol* **208**, 283-97 (2015).
