# Supplementary material for: A short linear motif in scaffold Nup145C connects Y-complex with pre-assembled outer ring Nup82 complex
Source: Nat Commun. 2017 Oct 24;8:1107. doi: 10.1038/s41467-017-01160-9 (PMC5653651; doi:10.1038/s41467-017-01160-9)
Supplement: Supplementary file 3 — Description of Additional Supplementary Files [file 41467_2017_1160_MOESM3_ESM.pdf]

Description of Additional Supplementary Files

File Name: Supplementary Data 1
Description: Comma Separated Values file containing the entire crosslinking mass spectrometry data set including all crosslinks regardless of their ld-score.
